# Non-Communicable Chronic Diseases Among Residents of a Remote Settlement in the Transbaikal Region of the Russian Federation

**DOI:** 10.5195/cajgh.2019.338

**Published:** 2019-05-13

**Authors:** Oleg Gaisenok

**Affiliations:** 1 Research Center for Medical Forecasting and Analysis, Moscow, Russia; 2 United Hospital with Outpatient Department, Moscow, Russia

**Keywords:** Chronic Noncommunicable Diseases, Screening Programs, Vascular Stiffness, Circulatory System Diseases, Mortality

## Abstract

**Introduction:**

Over a quarter of the population of the Russian Federation resides in rural communities. However, the data on chronic disease rates in these communities are limited, which makes screening for chronic diseases extremely important. The aim of this study was to measure the prevalence of chronic noncommunicable diseases among residents of a remote settlement in the Transbaikal region, Russian Federation.

**Methods:**

A sample of residents from the Transbaikal region settlement was screened in August 2017. The screening included a survey to determine the frequency of self-reported chronic diseases as well as sociodemographic and behavioral risk factors. Additionally, vascular stiffness was measured by volumetric sphygmography using the VaSera-1500 device. Descriptive statistics have been used for data analysis.

**Results:**

126 residents were screened for this study. The prevalence of chronic non-communicable diseases and their risk factors were: hypertension (56.3%), gastrointestinal diseases (33.3%), chronic obstructive pulmonary disease (13.5%), smoking (35.7%), obesity (19.1%), and diabetes mellitus (6.3%). Pathological vascular changes typical of atherosclerosis were found by volumetric sphygmography in 17.5% of cases, with 5.5% of those cases corresponding to significant forms of severe peripheral atherosclerosis (ABI<0.9). An analysis of mortality causes for this settlement for 2016–2017 found that cardiovascular diseases accounted for over 50% of the total number of deaths.

**Conclusion:**

This study showed a high prevalence of detectable chronic non-communicable diseases and their associated risk factors. Cardiovascular diseases were the main cause of mortality. Active prevention programs and screenings are required to reduce the burden of chronic diseases in this region.

## Introduction

Cardiovascular diseases (CVD) are the most common cause of death in the world. In 2013, 32% (17.3 million) of all deaths worldwide, were associated with CVD.^1^ Many of these deaths were due to the lack of access to cardioprotective medications such as aspirin, statins, and antihypertensive drugs.

The burden of CVD is exacerbated in Russia by logistic issues of reaching remote geographic areas and the complexity required to coordinate the delivery of skilled medical assistance to individuals located hundreds of kilometers away from medical facilities. As of January 1st, 2018, the rural and urban population of Russia were 37.5 million and 109.3 million respectively.^2^ The majority of observational and epidemiological studies in Russia are based on urban populations because of easier access to the participants. This bias of focusing on urban populations, may underestimate the burden of CVD and their risk factors in rural Russia, where only a few studies have been conducted. Therefore, studies focusing on the morbidity and mortality of rural population in Russia are needed to fill this important research gap.

The study “Epidemiology of cardiovascular diseases in different regions of the Russian Federation” reported rates of mortality from circulatory system diseases (CSD) for 12 regions of Russia.^3^ In five regions, CSD mortality in men aged 25–64 years exceeded the national Russian Federation average of 573 per 100,000 men; three of the five regions had CSD mortality rates more than 600 per 100,000 men. Among women aged 25–64 years, three regions had CSD mortality rates, exceeding the average for the Russian Federation.^3^

The Epidemiology of Cardiovascular Risk Factors and Diseases in Regions of the Russian Federation Study (ESSE-RF) reported on the prevalence of major diseases in almost 22 thousand individuals across 13 regions of the Russian Federation between 2012 and 2013. The overall prevalence of cardiovascular diseases was as following: arterial hypertension - 43.0% (men - 47.9%, women - 39.6%); coronary artery disease - 7.9% (men - 8.0%, women - 7.8%); liver diseases - 36.1% (men - 27.5%, women - 41.3%); and diabetes - 3.9% (men - 3.7%, women - 4.0%).^4^ Based on 2013 standardized total mortality rates for men in the Russian Federation the highest mortality rate was in Far Eastern Federal District.^5^

**Figure 1:**
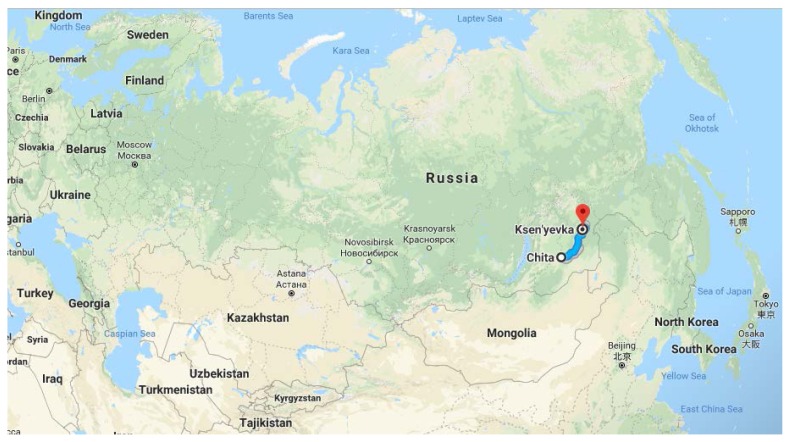
Ksenyevka’s location on the map of the Russian Federation

The Trans-Baikal region (Figure 1), which is apart of the Far Eastern Federal District, is one the least populated regions in Russia. The Trans-Baikal region was not included in the Russian epidemiological study of ESSE-RF, and there have been no recent specialized epidemiological studies analyzing the morbidity and mortality in this region. In addition, current comprehensive statistical data on CSD mortality are not available for Trans-Baikal’s rural and remote regions.

The primary objective of this study was to assess the prevalence of chronic noncommunicable diseases and their respective risk factors among residents of the remote settlement of Transbaikal region. Our second objective was to determine the prevalence of atherosclerosis and vascular diseases using volumetric sphygmography. To our knowledge, this is the first study in the past decades to assess chronic diseases in remote settlements of the Trans-Baikal region.

## Methods

### Study population

For this cross-sectional study, we surveyed Ksenyevka residents who applied for screening in August of 2017. Ksenyevka settlement is located in the Mogochinsky district of the Transbaikal region. It is 800 km away from the capital of the Trans-Baikal region, Chita (Figure 1), and 100 kilometers away from the nearest hospital. Residents were recruited through local advertisements. Inclusion criteria for our study were settlement residency and consenting to participate in the screening. No exclusion criteria were used. All participants gave written informed consent before participating in this study.

### Survey data

Data on sociodemographic, family history, and other risk factors for cardiovascular disease were collected. Data were also collected on gender, age, marital status, education, employment, disability, dietary habits, physical activity, smoking, and alcohol use. Presence of diseases, history of angina pectoris and myocardial infarction was assessed using Rose’s questionnaire.^6^

### Physical examination

Blood pressure was measured using a WelhAllyn tonometer (Germany) in accordance with the National Recommendations.^7^ Body weight measurements were performed using Maxwell electronic floor scales (China). Participants were measured once, without shoes and wearing light clothing. Height was measured using a stadiometer. Participants’ BMI were classified using the US Center for Disease Control criteria: underweight (BMI <18.5 kg/m2), normal weight (BMI 18.5 to <25kg/m2), overweight (BMI 25 to <30kg/m2), obese (≥30kg/m2), and obese class III (BMI ≥40kg/m2).^8^

### Cardiovascular assessments

Individuals were screened for the following cardiovascular diseases: arterial hypertension, angina pectoris, previous history of myocardial infarction and cerebral stroke, cerebrovascular diseases, valvular heart diseases, cardiac arrhythmias, and heart failure.

We measured vascular stiffness using the VaSera VS-1500 volumetric sphygmograph (Fukuda Denshi, Japan). The estimated vascular age of the patient (EVAP) was determined automatically by the device on the basis of a graphical transformation of the Cardio-ankle vascular index (CAVI) and the age of the patient. Date of birth was confirmed using the passport of each participant. The following health indicators were evaluated: cardiovascular vascular index on the right extremities (R-CAVI), estimated vascular age based on R-CAVI (R-CAVI age - EVAP), cardiovascular vascular index on the left extremities (L-CAVI), estimated vascular age based on L-CAVI (L-CAVI age - EVAP), ankle-brachial index (ABI) on the right extremities (R-ABI), and ankle-brachial index on the left extremities (L-ABI). The following generally accepted intervals were adopted as criteria for atherosclerotic vascular lesion: ABI 1.0—1.29 - norm, 0.91—0.99 - borderline condition, 0.41—0.9 - peripheral arterial disease of mild / moderate degree, <0.40 - severe peripheral arterial disease.^9^ Normative indicators of the CAVI index for the assessment of pathology were as follows: CAVI <8.0 - norm; 8.0—9.0 - borderline condition; > 9.0 - atherosclerotic lesion.^10^

The basic indicator for the assessment of the reduced left ventricle (LV) systolic function was an indicator PEP / ET> 0.36, which rises above this value with a decrease in LV systolic function.^11^ Based on the results of the screening, patients were provided health recommendations and referral to cardiologist.

### Mortality data

Mortality rates in Ksenyevka for 2016 and 2017 were obtained using death certificates from the local death registry and funeral home in Ksenyevka.

### Statistical analysis

Continuous variables were represented using means and standard deviations and categorical variables were listed as frequencies by absolute numbers and percentages.

## Results

126 residents were included in this study. This accounted for 4.6% of the total settlement population in 2017 (n=2717)^12^. The general demographic and anthropometric characteristics of patients are presented in Table 1. The screening revealed that only 37.3% (n=47) of the research participants had a normal body weight in accordance with WHO criteria.^8^ Almost three quarters of those surveyed had elevated blood pressure values (130/80 mmHg) at the time of the examination (74.6%; n=97). More than 25% (n=32) found out about the elevated blood pressure at the time of screening and were not aware of it prior to the study. Interviews and medical record reviews revealed that 20% of participants were not regularly taking their prescribed blood pressure medication at the time of screening. 35.2% patients with arterial hypertension (n=25) were aware of their disease diagnosis but were not aware which medications they were supposed to take. 76.2% (n=96) had reported having chronic disease diagnosis at the time of screening. The frequency of major chronic diseases among Ksenyevka residents included in this study are presented in Table 2. The most common diseases among the residents of Ksenyevka were arterial hypertension (56.3%), gastrointestinal diseases (33.3%), and COPD (13.5%).

Volumetric sphygmography data was collected from 124 residents (98.4%). Results for ankle-brachial index (ABI), cardiovascular vascular index (CAVI), estimated vascular age of the patient (EVAP), Weissler coefficient (PEP / ET) are presented in Table 3. 12.6% (n=16) of study participants had an EVAP greater than the maximum recorded true age and had disturbances in the systolic function based on PET/ET (Weissler’s coefficient). Based on the CAVI criterion for pathological changes in the vascular system, pathologies typical for atherosclerosis were detected in 17.5% (n=22) of cases, while in 5.5% (n=7) of patients had significant forms of severe peripheral atherosclerosis.

Mortality trends in Ksenyevka for 2016 and 2017 are presented in Table 4. CVDs were the main cause of mortality in this settlement. We noted that the contribution of cardiovascular diseases in the structure of mortality of the settlement slightly increased from 2016 (50%) to 2017 (65%). In 2016 cancer (20.5%) and suicide (13.6%) were the second and the third leading causes of mortality, whereas in 2017 second and third leading causes weere cancer and diabetes (8.6% each). The mortality rate from suicide was 13.6% in 2016 and 5.7% in 2017.

## Discussion

In this study we explored the prevalence of chronic non-communicable diseases and cardiovascular health status of residents of Ksenyevka, a remote settlement of the Trans-Baikal region, Russian Federation. Few studies report on the rural settlements of Trans-Baikal region, which has the second highest standardized total mortality rate among men in Siberian Federal District.^5^

In this study, we identified that the most prevalent diseases were arterial hypertension (56.3%), gastrointestinal diseases (33.3%), and COPD (13.5%). One quarter of patients with arterial hypertension did not receive the therapy prescribed by the doctor. Almost three quarters of those surveyed had elevated blood pressure values. 19.1% of study participants were obese and 41.2% were overweight. We found that 6.3% of screened participants suffered from diabetes mellitus, 5.5% from coronary artery disease, and 6.3% and 4.0% had previous history of myocardial infarction and cerebral stroke respectively. We used volumetric sphygmography to evaluate vascular stiffness and identify preclinical and clinical atherosclerosis. Volumetric sphygmography is a simple and convenient method for screening programs that has been widely used in observational studies.^13^–^16^ In our study, pathological vascular changes typical for atherosclerosis were detected in 17.5% of cases, with 5.5% of them having signs of severe stenotic atherosclerosis.

Reducing the incidence of CVD and its complications in Russia will require the introduction of preventive programs both at the population and state institutions level.^17^ Non-pharmacological preventive programs, aimed at modifying known risk factors, is a critical part of reducing the burden of CVD. The North Karelia project in Finland aimed at promoting healthy lifestyles, resulted in a significant reduction in blood pressure and total cholesterol in the population, as well as a reduction in the percentage of smokers from 52% to 32%. This led to a reduction in mortality rates from coronary heart disease in Finland by 65%^18^.

Anticoagulants and statins are effective drugs cardiovascular disease, in which low adherence is a problem.^19^,^20^ We have found that many patients have low adherence to prescribed treatment and do not follow the doctor’s recommendations. One of the reasons for the low adherence to taking statins is the perceived lack of clinical effect by the patients.^21^ Preventive population screening programs are an effective tool that allows timely detection CVD in patients, as well as informs necessary treatments and motivates healthy lifestyle.^13^,^14^

Data on the prevalence of diseases among those surveyed in Ksenyevka showed higher prevalence of hypertension and diabetes compared to ESSE-RF study, whereas the prevalence of CAD and gastrointestinal diseases was lower.^4^ Interestingly, vascular age of patient was higher than the patient’s chronologic age in 23.2% of screened Moscow residents compared to 12.3% of screened participants in Ksenyevka^14^. Sumin A.N. et al reported the 16.6% prevalence of type 2 diabetes mellitus in Western Siberia^22^. The authors also reported that CAVI increase in a population sample of Western Siberia associated with type 2 diabetes.^22^ Interestingly, the suicide rate has decreased most than two times in 2017 compared to 2016. Suicide prevention should be an important priority of the future intervention programs.

There were a few limitations for our study. First, study used self-report for the identification of several chronic diseases. Second, we surveyed only a small percentage of the settlement population (4.6%). The low recruitment may reflect the lack of motivation of the community to improve health in our study population or short recruitment period. Assessment period was 3 days which was another potential reason for the small turn-out. Third, there was a risk of volunteer and healthy worker bias, as 92% of the participants were of working age, which may not be reflective of the entire settlement. This study did not collect data on participant nationality; however, the overwhelming majority self-identify as Russians.^23^ This study did provide a general depiction of the major clinical conditions of Ksenyevka, which has never been previously reported in scientific medical journals.

Given the high prevalence of cardiovascular disease in our study and previous data on cardiovascular disease prevalence in the Russian Federation, we suggest that health care efforts emphasis preventative strategies especially in the remote regions. Regular simple screening programs for cardiovascular risk factors would also assist in efforts to increase patient adherence to treatment regimens, to monitor health status, and to maintain a healthy lifestyle.^24^,^25^

The study reported a large percentage of detectable chronic non-communicable diseases and risk factors in a remote settlement population in the Trans-Baikal region of the Russian Federation. Cardiovascular diseases were the main cause of mortality. Our findings support the need for preventive screening programs to effectively identify chronic non-communicable diseases and reduce know risk factors for these diseases.

## Figures and Tables

**Table 1:** General characteristics of study participants

Characteristics	% (n)
Age, yrs^1^	45.3 ± 14.2
Male	51.6% (65)
Current Smokers	35.7% (45)
Mean blood pressure (BP), mm Hg (in the group)	146.0/92.5 ± 23.4/12.3
BP at the time of the examination:	
more than 130/80 mm Hg	74.6 % (97)
more than 140/90 mm Hg	52.4% (66)
BMI, kg/m^2^	27.4 ± 5.9
Underweight (BMI <18.5 kg/m^2^)	2.4% (3)
Lean (BMI: 18.5 to <25 kg/m^2^)	37.3% (47)
Overweight (BMI = 25 to <30 kg /m^2^)	41.2% (52)
Obese (BMI> 30 kg /m^2^)	19.1% (24)
Obese III (BMI> 40 kg /m^2^)^2^	6.3% (8)
Currently Employed	92.0% (116)

1 Continious variables are listed as mean ± standard deviation

2 Is this a subset of the obese group.

**Table 2:** Prevalence of self-reported non-communicable conditions

Disease	Frequency (%)	n
Arterial hypertension	56.3	71
Gastrointestinal diseases	33.3	42
COPD or bronchial asthma	13.5	17
Heart rhythm disturbances (including atrial fibrillation)	8.7	11
Diabetes mellitus	6.3	8
Previous history of myocardial infarction	6.3	8
Coronary artery disease	5.5	7
Neoplasms	5.5	7
Heart valve diseases	4.0	5
Previous history of cerebral stroke	4.0	5
Psoriasis	2.4	3
Nephritis	2.4	3
Severe sleep apnea	2.4	3
Moshkovsky-Shaffar hereditary hemolytic anemia	1.6	2
Rheumatic diseases	1.6	2
Thrombophlebitis	0.8	1
Tuberculosis	0.8	1
Epilepsy	0.8	1

**Table 3:** Volumetric sphygmography vascular characteristics

Clinical Parameter	Mean ± SD	Normal Range	% of patients outside of normal range
ABI (right)	1.07 ± 0.09	>1.00	5.5
ABI (left)	1.07 ± 0.02	>1.00	4.1
CAVI (right)	7.39 ± 1.45	<8.0	15.1
CAVI (left)	7.40 ± 1.51	<8.0	17.5
EVAP (right)^1^	43.4 ± 17.9	<71 yrs	11.0
EVAP (left)^1^	43.4 ± 18.5	<71 yrs	12.6
PET/ET	0.32 ± 0.05	<0.36	12.6

1 Reference value was determined from the oldest true age of the study participants

**Table 4:** January 2016 to September 2017 mortality rates by clinical indication for the Ksenyevka population

Cause of death	2016 % (n)	2017 % (n)
Cardiovascular diseases	50.0 (22)	65.0 (23)
Cancer	20.5 (9)	8.6 (3)
Cirrhosis	0.0 (0)	2.8 (1)
Diabetes mellitus	2.3 (1)	8.6 (3)
Infection	0.0 (0)	2.8 (1)
Suicide	13.6 (6)	5.7 (2)
Tuberculosis	0.0 (0)	5.7 (2)

Total	100 (44)	100 (35)

## References

[1] (2017). Heart Disease and Stroke Statistics-2017 Update: A Report from the American Heart Association. Circulation.

[2] Estimate of the number of resident population on January 1, 2018 and an average for 2017.

[3] Shalnova SA, Konradi AO, Karpov Yu A, Kontsevaya AV, Deev AD, Kapustina AV, Khudyakov MB, Shlyakhto EV, Boytsov SA (2012). Cardiovascular mortality in 12 Russian Federation regions – participants of the “Cardiovascular Disease Epidemiology in Russian Regions” study. Russ J Cardiol.

[4] Shalnova SA, Oganov RG, Deev AD, Imaeva AE, Lukyanov MM, Artamonova GV, Gatagonova TM, on behalf of the ESSE-RF study work team (2015). Comorbidities of ischemic heart disease with other non-communicable diseases in adult population: age and risk factors association. Cardiovascular Therapy and Prevention.

[5] Kolesnikov SI, Savilov ED, Savchenkov MF, Leshchenko Ya A, Malov IV, Anganova EV, Astaf’ev VA, Shugaeva SN (2016). Sanitary-Epidemiological Status of Siberian Population (Medico-Demographical and Epidemiological Characteristics). Annals of the Russian Academy of Medical Sciences.

[6] Rose GA, Blackburn H, Gillum RF, Prineas RJ (1982). Cardiovascular survey Methods.

[7] Chazova IE, Ratova LG, Boitsov SA, Nebieridze DV (2010). Recommendations for the management of arterial hypertension Russian Medical Society of Arterial Hypertension and Society of Cardiology of the Russian Federation. System Hypertension.

[8] Defining Adult Overweight and Obesity.

[9] (2011). ESC Guidelines on the diagnosis and treatment of peripheral artery diseases: Document covering atherosclerotic disease of extracranial carotid and vertebral, mesenteric, renal, upper and lower extremity arteries: the Task Force on the Diagnosis and Treatment of Peripheral Artery Diseases of the European Society of Cardiology (ESC). Eur Heart J.

[10] Namekata T, Suzuki K, Ishizuka N, Shirai K (2011). Establishing baseline criteria of cardio-ankle vascular index as a new indicator of arteriosclerosis: a cross-sectional study. BMC Cardiovasc Disord.

[11] Milyagin VA, Milyagina IV, Purygina MA, Osipenkova TA (2014). The method of volumetric sphygmography on the device Vasera VS-1500N: guidelines.

[12] The population of the Russian Federation for municipalities on January 1, 2017- published on July 31, 2017.

[13] Gaisenok OV, Dorokhov SI, Kalashnikov SV, Leonov AS, Vlasova LA (2017). The value of population-based programs within “Healthy Heart Day” moves to identify hypertension and major risk factors for cardiovascular diseases. Profilakticheskaya meditsina.

[14] Gaisenok OV, Medvedev PA, Trifonova SS, Shatalova IV, Martsevich SY, Sidorenko BA (2015). Application of CAVI Index in Clinical Practice: Calculated Vascular Age as a Tool for Decision on Additional Examination of Patients With Cardiovascular Diseases. Kardiologiia.

[15] Rogoza AN, Kaveshnikov VS, Trubacheva IA, Serebriakova VN, Zairova AR, Zhernakova YV, Oshepkova EV, Karpov RS, Chazova IE (2014). Vascular Wall in Adult Population of Tomsk in the Framework Of The Project Essay RF. Systemic Hypertension.

[16] Sumin AN, Shcheglova AV, Fedorova NV, Artamonova GV (2015). Values of Cardial-Ankle Vascular Index in Healthy People of Different Age by the Data of Esse-RF Study in Kemerovskaya Region. Cardiovascular Therapy and Prevention.

[17] The Russian Society of Cardiology. The Russian National Atherosclerosis Society. Russian Society of Cardiosomatic Rehabilitation and Secondary Prevention (2017). Diagnostics and correction of lipid metabolism disorders in order to prevent and treat atherosclerosis. Russian recommendations VI revision. Journal of Atherosclerosis and Dyslipidaemias (JAD).

[18] Jousilahti P, Laatikainen T, Salomaa V, Pietilä A, Vartiainen E, Puska P (2016). 40-Year CHD Mortality Trends and the Role of Risk Factors in Mortality Decline: The North Karelia Project Experience. Glob Heart.

[19] Gaisenok O, Martsevich S, Tripkosh S, Lukina Y (2015). Analysis of lipid-lowering therapy and factors affecting regularity of statin intake in patients with cardiovascular disease enrolled in the PROFILE registry. Rev Port Cardiol.

[20] Gaisenok Oleg V, Leonov Anton S (2017). Therapy in the Prevention of Thromboembolic Complications in Patients with Atrial Fibrillation: Prospects for Higher Appointment of New Oral Anticoagulants in Clinical Practice. Cardiology and Cardiovascular Research.

[21] Wei MY, Ito MK, Cohen JD, Brinton EA, Jacobson TA (2013). Predictors of statin adherence, switching, and discontinuation in the USAGE survey: understanding the use of statins in America and gaps in patient education. J Clin Lipidol.

[22] Sumin AN, Bezdenezhnykh NA, Fedorova NV, Bezdenezhnykh AV, Indukaeva EV, Artamonova GV (2018). The Relationship of Visceral Obesity and Cardio-Ankle Vascular Index with Impaired Glucose Metabolism According to the Esse-RF Study in West Siberian Region. Klin Med (Mosk).

[23] (2017). National composition of Russia.

[24] Schwalm JD, McKee M, Huffman MD, Yusuf S (2016). Resource Effective Strategies to Prevent and Treat Cardiovascular Disease. Circulation.

[25] Yusuf S (2016). Why do people not take life-saving medications? The case of statins. Lancet.

